# A 2D, 3D and text dataset of fictional forensic scenes (FFS)

**DOI:** 10.1016/j.dib.2026.112825

**Published:** 2026-05-07

**Authors:** Hervé Daudigny, Pierre Grussenmeyer

**Affiliations:** a School of Criminal Justice, University of Lausanne, Batochime, 1015 Lausanne, Switzerland; b Université de Strasbourg, INSA Strasbourg, CNRS, ICube Laboratory UMR 7357, Photogrammetry and Geomatics Group, 67000, France

**Keywords:** Digital twin, Forensic scene, Images, 3D, Terrestrial scanner laser

## Abstract

In this article, we present a dataset of Fictional Forensic Scenes (FFS) with the aim of providing opportunities for processing rare data. Access to real data from actual scenes is difficult due to the confidential and sensitive nature of investigations. The dataset is provided in formats compatible with conventional processing as well as AI-based workflows. These data can be used for multiple purposes including scientific research, educational training, and operational applications. The dataset covers Fictional Forensic Scenes (less than 30 meters wide), which staged at the School of Criminal Justice (ESC, Ecole des sciences criminelles) of the University of Lausanne. For each scene, data has been acquired using a terrestrial laser scanner (TLS), producing 3D point clouds and panoramic views. Photographs documenting the scenes and a structured record of observed elements and associated traces have been produced for each scene. (Daudigny & Grussenmeyer, 2024).

Specifications Table

**Table:** 

Subject	Computer Sciences
Specific subject area	Support the creation of 3D, 2D, and text-based processing in the field of forensic scenes by providing a dataset compatible with standard formats.
Type of data	Images (.png) et (.jpeg), 3D point clouds (.las), Text Files (.xlsx)
Data collection	The 3D data were acquired using a terrestrial laser scanner (Faro Premium 70) with the following settings: resolution ¼, quality 2 (same device settings as those used in Daudigny & Grussenmeyer [1]). This corresponds to a measurement accuracy of 6mm at 10m and an acquisition of 43.7 million points (10240 * 4267 points). This laser scanner allows the creation of a panoramic view. Color mode was activated so that 3D point clouds and panoramic views are available in black-and-white and in color. A single scan is performed for each digitized scene. Photographs documenting the scenes are taken using a CATS60 smartphone sensor (resolution 3120*4160, focal length 3.65mm), except for scenes th_0067 to th_0073, which were captured with a Canon EOS 60D (5184*3456, focal length 17-85 mm).
Data source location	School of Criminal Justice (ESC, Ecole des Sciences Criminelles), University of Lausanne ((46.51995250909327, 6.5723435545936875) City/Town/Region: Lausanne, canton of Vaud Country: Switzerland
Data accessibility	Repository name: Fictional Forensic Scene (FFS) on Zenodo Data identification number: Part1 (th_0001-th_0020): https://doi.org/10.5281/zenodo.17151982 Part2 (th_0021-th_0040), https://doi.org/10.5281/zenodo.17164106 Part3 (th_0041-th_0060): https://doi.org/10.5281/zenodo.17164834 Part4 (th_0061-th_0080): https://doi.org/10.5281/zenodo.17165128 Part 5 (th_0081-th_0100): https://doi.org/10.5281/zenodo.18623225
Related research article	Daudigny, H., & Grussenmeyer, P. [[1], December]. A dataset to test AI on forensic scenes. In 8th International ISPRS Workshop LowCost 3D-Sensors, Algorithms, Applications (Vol. 48, pp. 117-123).

## 1 Value of the Data

• A wide range of detailed data: The dataset consists of 100 Fictional Forensic Scenes (FFS) but this number is expected to grow in the future, as new acquisitions are ongoing and will be disseminated as they become available. For each scene, three types of data are available: 3D data (3D point clouds) from terrestrial laser scanning [2], 2D image data (panoramic views from terrestrial laser scanning, scene documentation images), and text data (lists of exhibits, scene summary tables).
• Rare and confidential data: Real forensic scenes are generally inaccessible for public dissemination due to their sensitive and confidential nature. Furthermore, the data that may be accessible usually consists of a single fictional scene, which does not allow for varied processing and testing in different scenarios [[3], [4], [5]].
• Data processing with classic algorithms or AI: This dataset is well suited for classic algorithms or AI-based processing, such as machine learning (ML), deep learning (DL) [1]. 2D and 3D data can be used to test labelling or self-labelling. 3D point clouds can also be useful for testing filtering and cleaning tools. In ML, a practical example could be the detection of markers and their numbering. In DL, semantic segmentation tasks could be carried out. 3D data (.las) can be opened and processed using CloudCompare software, as well as using Open3D, for example. In 3D, segmentation could be used to detect planes (morphological analysis of bloodstains), the 3D position of objects (bodies, weapons, markers); this also applies to the scene’s environment (floor, wall, ceiling, etc.). In 2D, both images of documentation and panoramic images can be used to support segmentation to detect the same features. This type of dataset can be integrated directly or, following processing, into 3D or 2D processing workflows, either on its own or alongside other datasets. Furthermore, tests can be carried out using 2D methods (OpenCV, Yolo, etc.) or 3D processing techniques (RANSAC, PointNet++, etc.). The files are in standard formats (.las, .png, .jpg, .xlsx), making them easily interoperable with processing pipelines. 3D point clouds are perfectly divisible, which multiplies the processing possibilities. The image data can also be used in these areas of application, as can the text data contained in the inventories of exhibits. This dataset can therefore be used by focusing on specific subsets or, conversely, by leveraging the full range of available data (3D point clouds, panoramic images, documentation images, texts).
• Creating a digital twin: This dataset, serving as a digital twin of the scenes, can also be used for education, communication, and operational training. The data is compatible for integration with other systems, such as virtual reality headsets that support 3D or virtual tours, for example. The various data can also be used for image processing and 3D data analysis by students or professionals. These digital twins can also support forensic science domains, such as ballistics [6].

## 2 Background

Forensic scene investigation and reconstruction remain a highly specialized area with limited publicly accessible data. As a result, opportunities for training or communication, particularly for professionals (magistrates, investigators, etc.), are constrained by the confidentiality of data captured on real scenes. This dataset is therefore intended as a repository of fictional scenes that are as close as possible to real scenes and can be easily used in either 3D or 2D. In addition, this dataset is useful in the context of automated data processing [1].

This article and the one by Daudigny & Grussenmeyer [1] form part of the same research project on the digitisation of crime scenes. The first article focuses on methodology and experimentation; it explains how the initial data were generated and how they were tested using machine learning and deep learning approaches. The second article builds on this work to publish and describe the dataset itself, highlighting its structure, scope, accessibility and reusability. The second article therefore follows on from the first: one presents the research findings, whilst the other provides the data as a resource.

Currently, when a crime scene or accident scene is processed by forensic specialists, the scene is secured. Numerous traces (cartridge cases, bullets, bodies, reddish traces, etc.) are searched, detected, located, documented, collected and secured [7]. Accurately positioning the traces in relation to one another and to the general environment is fundamental to understanding and reconstruct the sequence of events at the scene. It is therefore easy to become overwhelmed by the complexity of assessing a scene and the large number of traces to be integrated and represented in a 2D and 3D environment. Assessing a scene requires real forensic expertise [8].

Performing all these description (attribute data) and positioning operations manually is time-consuming and potentially error prone. Semi-automated tools exist and are sometimes implemented, but they require significant processing power and domain knowledge [9]. Thus, in an era where artificial intelligence is developing and enabling the automation of tedious tasks, this dataset provides an initial basis for testing machine learning or deep learning approaches that can simplify the work of forensic technicians or specialists [1].

## 3 Data Description

The dataset includes fictional crime scenes or accident scenes called Fictional Forensic Scenes (assault, burglary, drug trafficking, etc.). Please note that some scenes may be disturbing to an unsuspecting audience, as some mannequins are very realistic. The scenes were selected so that they did not extend more than 30 meters on either side of the scan position to avoid complicating subsequent processing. Each scene was captured with a terrestrial laser scanner, photographed and documented with notes on the forensic traces and exhibits observed.

Each scene was named to facilitate data retrieval, following the format “th_XXXX,” where “XXXX” corresponds to the scene number. The suffixes _coul and _nb refer to colour and black-and-white data respectively, whilst _full refers to ‘full’ resolution. Text data is provided in two languages (extension _ENG for english, _FR for french).

On Zenodo, scenes are grouped in packages (th_0001-th_0020, th_0021-0040, th_0041-0060, th_0061-0080, th_0081-0100), generally named th_XXXX-YYYY. Future new scenes will be integrated into linked repositories.

For each packages, the dataset is organized into a structure of folders:

• “description” contains two main spreadsheets describing the scenes in English and French (named th_XXXX-YYYY_FR.xlsx and Forensic_th_XXXX-YYYY_ENG.xlsx). These spreadsheets list the key details of the scenes, such as the scene type, the number of markers and their numbers, the overall traces, etc.;
• “img” holds documentation photographs for each scene (the number of documentary images may vary but averages 12.2 images for the first 100 scenes. A very small number of scenes do not include markers or documentary photographs (5 out of the first 100 scenes: th_0045, th_0047, th_0049, th_0051, th_0051) or documentary images (9 out of the first 100 scenes: th_0001, th_0032, th_0033, th_0034, th_0039, th_0042, th_0061, th_0080, th_0081). These scenes are highlighted in blue and yellow respectively in the main spreadsheets). The .jpg images are in Baseline format with a quality setting from 95 to 98 (form more information see Table 3);
• “IPC” (List of exhibits) contains two identical spreadsheets (only the language is different) listing the traces and exhibits for each scene named th_XXXX_FR.xlsx and th_XXXX_ENG.xlsx;
• “pano” includes black and white panoramic images (named pano_th_XXXX_nb.jpg);
• “scan” contains laser scanner data (3D point clouds named th_XXXX_nb.las and th_XXXX_coul.las) and panoramic images in black-and-white (pano_th_XXXX_nb.jpg) and in colour (pano_th_XXXX_coul.jpg and pano_th_XXXX_coul_full.png).
  *jpg images are in Baseline format with a quality level of 50, whilst .png images are non-interlaced and have a colour depth of 8 bits.*
  *3D and 2D data has not been filtered, cleaned, annotated or labelled, to give users greater freedom.*

**Fig. 1:**
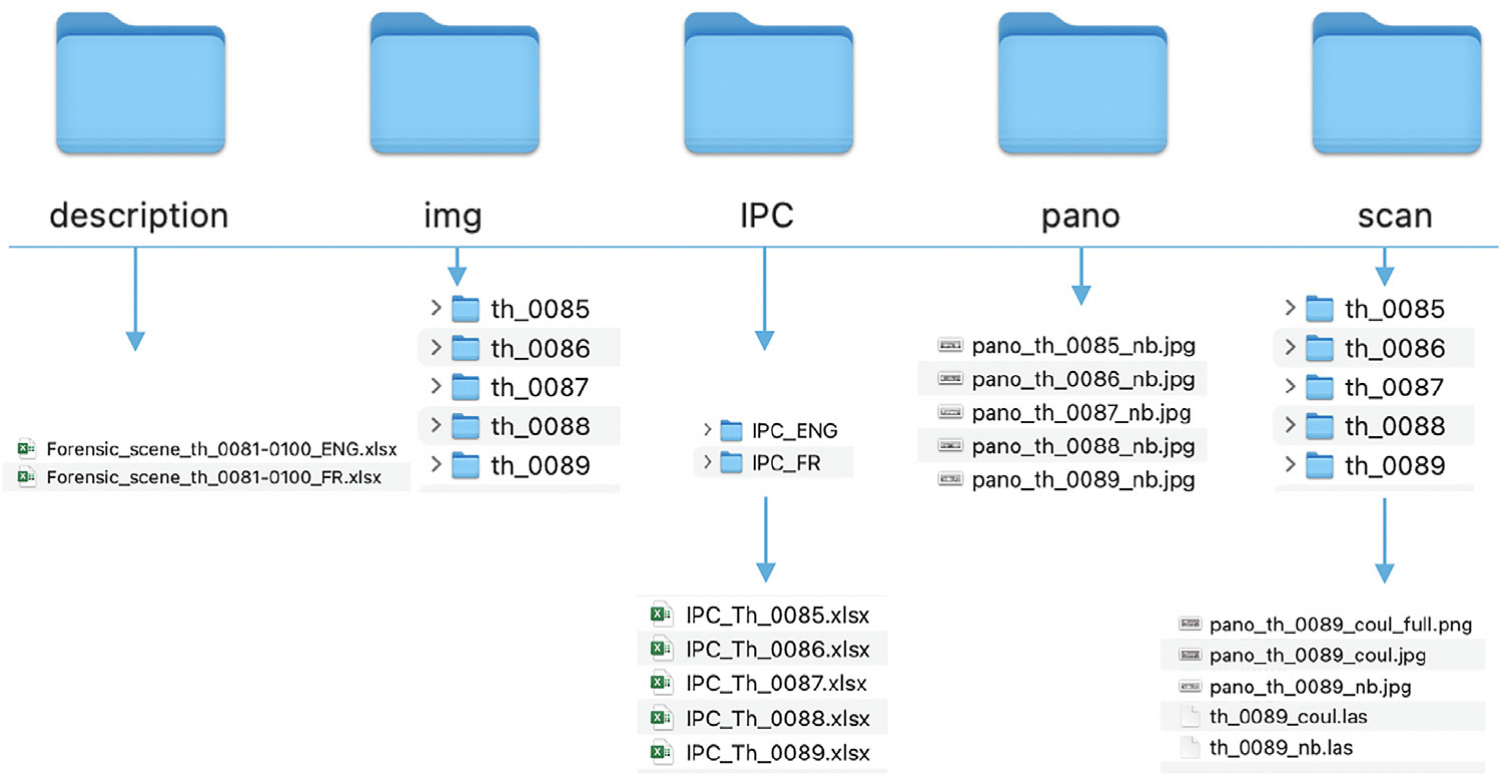
Details of data organization, available in the README file on Zenodo repositories (see SPECIFICATIONS TABLE /Data accessibility).

Further information on the data organization and description can be found in Table 1, 2 and Fig. 1.

**Table 1:** Structure and files by scene, (average calculated using the first 100 scenes).

Type	Folder	Name	Formats	Number of files	Average size / file in MB
Photographs (documentation)	img/th_XXXX	Automatic naming by the device	.jpg	It depends on the scene. On average: 12,2	3,1
List of exhibits in french	IPC/IPC_FR	IPC_Th_XXXX	.xlsx	1	0,01
List of exhibits in english	IPC/IPC_ENG	IPC_Th_XXXX	.xlsx	1	0,01
Panoramic images in black-and-white	Pano	pano_th_XXXX_nb	.jpg	1	6,7
Panoramic images in black-and-white	scan/th_XXXX	pano_th_XXXX_nb	.jpg	1	6,7
Panoramic images in colour	scan/th_XXXX	pano_th_XXXX_coul	.jpg	1	2
Panoramic images in colour	scan/th_XXXX	pano_th_XXXX_coul_full	.png	1	361,9
3D point cloud in black-and-white	scan/th_XXXX	th_XXXX_nb	.las	1	1’158,2
3D point cloud in colour	scan/th_XXXX	th_XXXX_coul	.las	1	840,6
			Total in average	20,2	2,4 GB

**Table 2:** Characteristics for the first 100 scenes.

Per the 100 first scenes:	Number	Formats	Total size in MB
Photographs (documentation)	1220	.jpg	3’831
Text (lists of exhibits)	200	.xlsx	2
Panoramic Images	400	.jpg or .png	37’730
Point clouds	200	.las	199’880
Text (General description)	200	.xlsx	2

The “pano” folder allows an overview of the scenes through black-and-white visual representation. Each scene contains two black-and-white panoramic images (identical images: one in the pano folder and the other in the scan folder, named pano_th_XXXX_nb.jpg).

For the first 100 scenes, the dataset comprises 2,220 files with a total size of 241 GB (calculated using Table 2).

## 4 Experimental Design, Materials and Methods

Some of the Fictional Forensic Scenes that makes up the dataset consist of scenes prepared for practical education sessions in crime scene and complex cases investigation for students of the School of Criminal Justice, University of Lausanne. Additional scenes were created specifically for this project to enrich the dataset. These scenes include traces commonly found at real crime scenes (mannequin bodies, ballistic exhibits, reddish traces, objects, etc.). For each scene, distinctive evidence indicators (typically polygonal in shape and visually contrasted to the scene) were positioned to reference each identified trace. These scenes can be situated indoors or outdoors.

As with operations carried out on a real scene of investigation, documentation photographs are taken. These were captured using the sensor of a CATS60 smartphone, except for scenes th_0067 to th_0073, which were acquired with a Canon EOS 60D DSLR camera because the sensor was not available [1]. Further information on these devices can be found in Table 3.

**Table 3:** Characteristics of photographic sensors, based on Table 4 in Daudigny & Grussenmeyer [1].

Sensor	Resolution	Bands	Focal length	Average exposure	Format / Quality
CatS60	3120*4160	RGB	3,65mm	1/20	.jpg (Baseline) / 98
Canon EOS 60D	5184*3456	RGB	17- 85mm	1/30	.jpg (Baseline) / 96

Depending on the shooting conditions, the exposure time and aperture may vary; this is why Table 3 shows the average exposure time. All images are converted directly by the digital cameras to .jpeg format, with the quality settings also shown in Table 3.

In addition to the information shown in Table 3, the cameras were configured using different settings. For the CATS60, the following settings were applied: automatic mode, auto ISO, ‘Superfine’ image quality, flash off, no location data recorded, and no digital zoom used. For the Canon EOS 60D, the following settings and lens were used: image stabilisation, autofocus, Flash off, EFS 17–85mm lens.

**Fig. 2:**
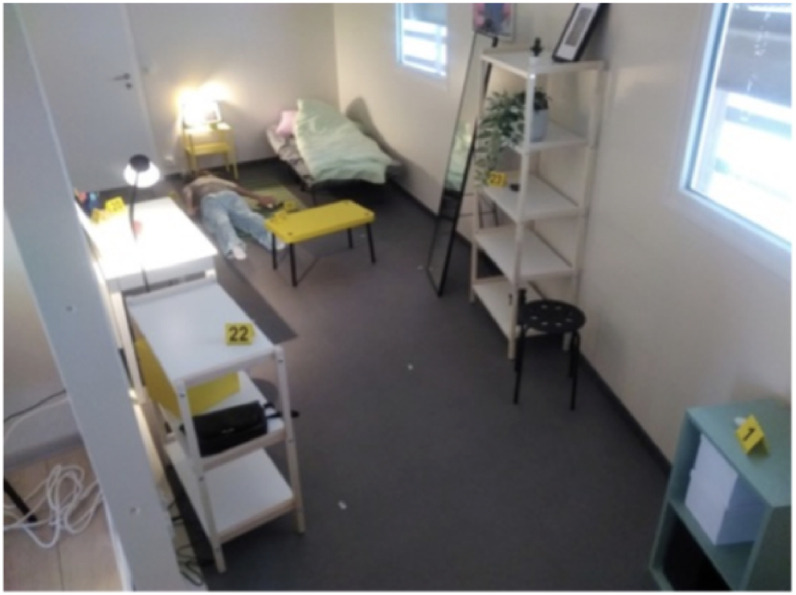
General view (available at th_0061_0080/img/th_0063/IMG_20250326_123223.jpg).

**Fig. 3:**
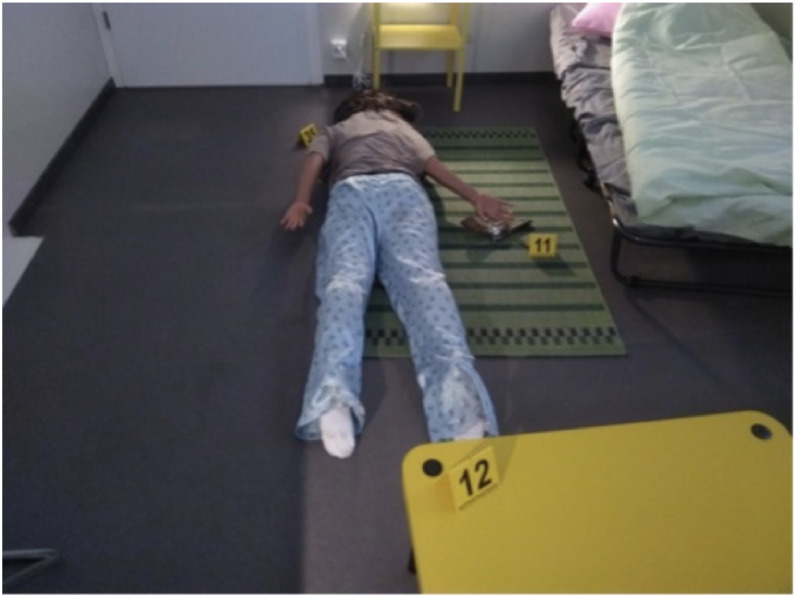
Close-up allowing to follow a path (available at th_0061_0080/img/IMG_20250326_123932.jpg).

**Fig. 4:**
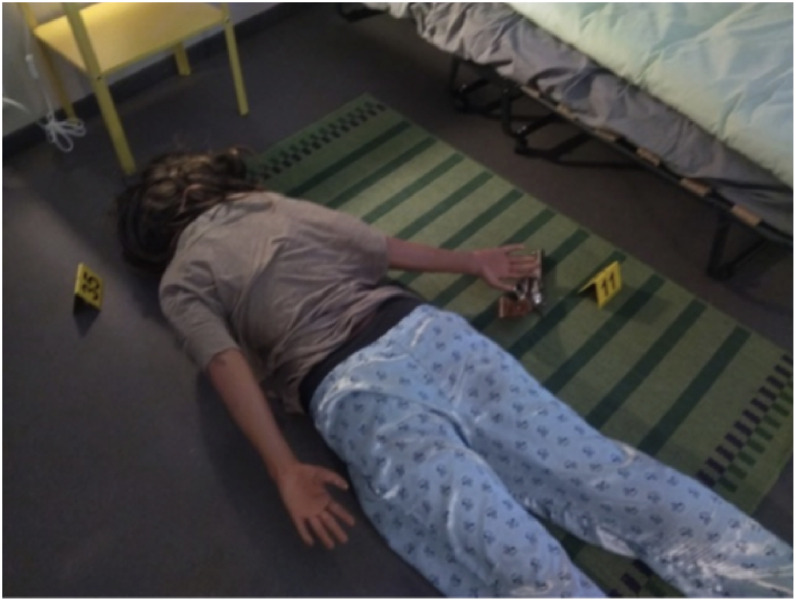
Close-up shot (available at th_0061_0080/img/IMG_20250326_123938.jpg).

These photographs, taken from general to specific, allow a comprehensive understanding of the scene. To take documentary photographs, the following procedure is generally followed (where the layout of the site allows). First, a wide-angle photograph must be taken from the four corners of the scene (see Fig. 2), followed by close-up photographs of areas containing markers (see Fig. 3, Fig. 4). It is necessary to follow a specific route to orientate oneself within the scene and to be able to locate each marker in relation to its nearest neighbours. Daudigny & Grussenmeyer [1].

**Fig. 5:**
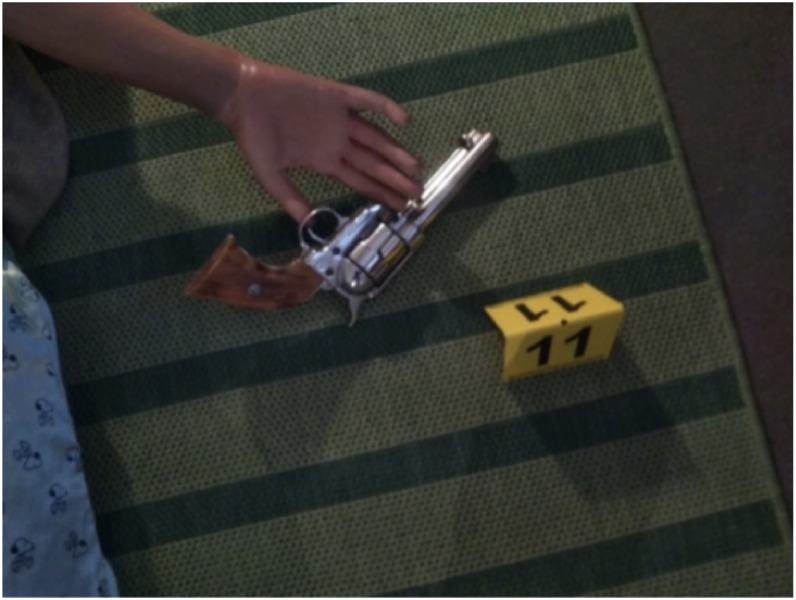
*Detailed shot (*available at th_0061_0080/img/th_0063/IMG_20250326_123944.jpg).

To do this, you need to decide on a route through the scene; you usually start at one side and work your way across. Once all the areas containing markers have been photographed, you should take close-up photographs of each trace, ensuring that the relevant marker is visible in the photograph (see Fig. 5) [7].

**The folder th_0061_0080 is available on Zenodo repositories (see SPECIFICATIONS TABLE /Data accessibility/Part4 (th_0061-th_0080)).**

**Fig. 6:**
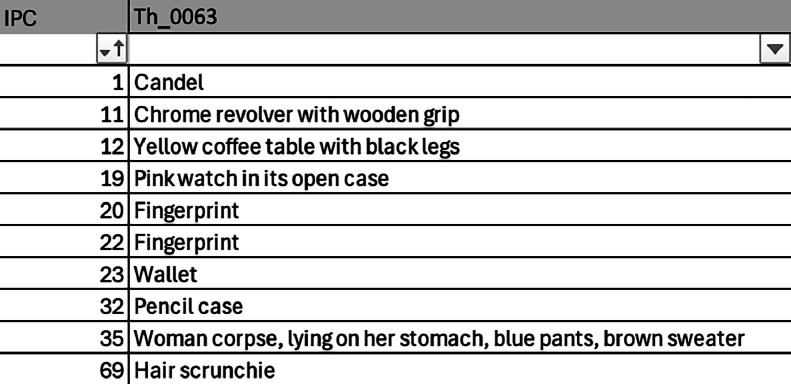
List of exhibits for scene th_0063 (available at th_0061_0080/IPC/IPC_ENG IPC_Th_0063.xlsx on Zenodo repositories (SPECIFICATIONS TABLE /Data accessibility/Part4 (th_0061-th_0080).

Notes are also taken directly at the scene, and subsequently transcribed into an inventory spreadsheet of exhibits, listing the index numbers and descriptions of the traces found (see Fig. 6). Such documentation is particularly useful as it is generally included in legal proceedings.

All scenes were 3D captured with a Faro Premium 70 terrestrial laser scanner, belonging to the School of Criminal Justice [10]. It was used with the following settings: resolution ¼, quality 2. Placed on a tripod, the device acquired approximately 43.7 million points (10,240 * 4,267 points) during its first half-turn. With the color setting selected, the laser scanner acquired a panoramic color image during its second half-turn.

When scanning a scene, a single scan is performed using the laser scanner. Each scan is positioned within the scene, taking care to minimise obscured areas and ensure that elements of interest (such as body) are not cut off. The laser scanner is also positioned to maximise the number of markers visible from the acquisition point. The optical centre of the laser scanner is placed at a height of between 1.50 m and 1.70 m above the floor of the scene.

As each scene contains only a single scan, no registration is performed. Although reference markers (such as spheres or checkerboards) may appear, these are not used.

### 4.1 *Data pre-processing*

After laser scanning the raw data from were processed using Faro Scene software (version 2023.0.1.10677). The point cloud is not modified, except to remove elements containing personal data (number plates, visible people, etc.) and to colour it. There is therefore no noise reduction, nor is any infrastructure (ceiling, wall, etc.) removed. As indicated in the “Data Description” section, no georeferencing is carried out; the coordinates are expressed in a local coordinate system.

**Fig. 7:**
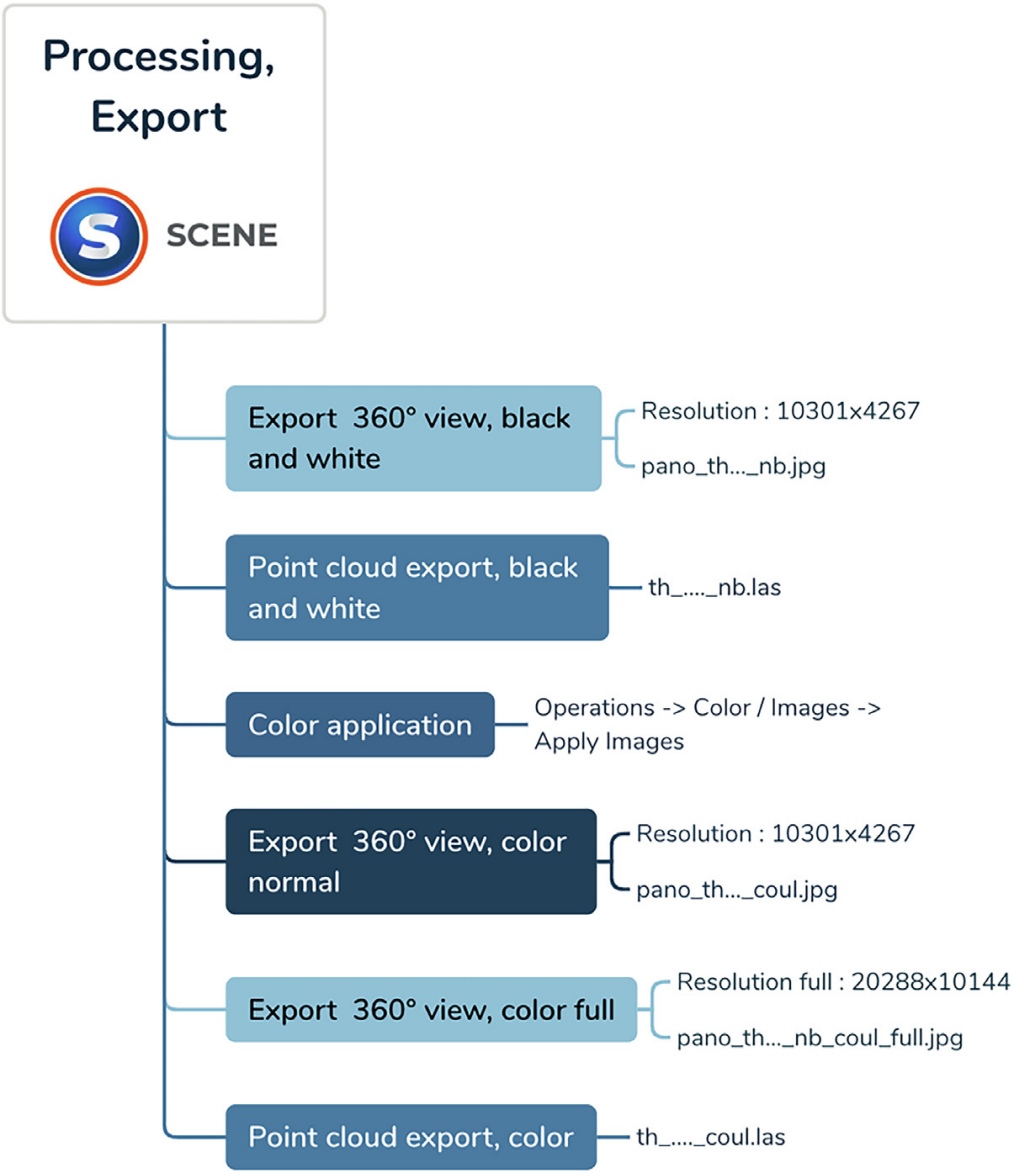
Details of processing and export operations for terrestrial laser scanner data, available on Zenodo in the Readme file on each repository (see SPECIFICATIONS TABLE /Data accessibility)

**Fig. 8:**
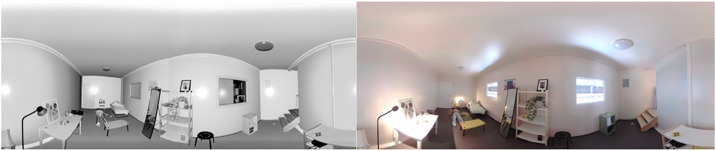
Example of black-and-white and colour panoramic views (available at th_0061_0080/scan/th_0063, files: pano_th_0063_nb.jpg and pano_th_0063_coul.jpg) on Zenodo repositories (see SPECIFICATIONS TABLE /Data accessibility/Part4 (th_0061-th_0080)).

**Fig. 9:**
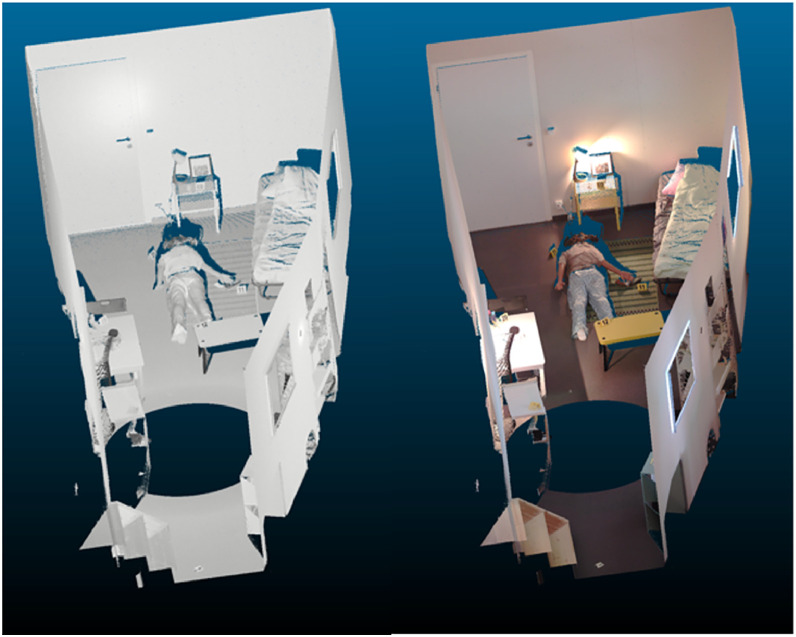
Example of black-and-white and 3D point cloud view (available at th_0061_0080/scan/th_0063, files: th_0063_coul.las and th_0063_nb.las on Zenodo repositories (SPECIFICATIONS TABLE /Data accessibility/Part4 (th_0061-th_0080).

3D cloud coloring was applied. Black and white (nb) and color (coul) exports were then made to obtain three panoramic image files (pano_th_XXXX_nb.jpg, pano_th_XXXX_coul.jpg, pano_th_XXXX_coul_full.jpg), and two 3D files (th_XXXX_nb.las, th_XXXX_coul.las) per scene (see Figs. 7, Fig. 8, Fig. 9).

All data and figures included in the article are available on Zenodo, which hosts the dataset.

## Limitations

The dataset still has some limitations. The dataset is too limited to support alone the training of AI processing, particularly in terms of images (2D). Conversely, to be processed by AI, the 3D point clouds will likely require to be divided up in order to reduce processing resources. In addition, to perform segmentation, both images and 3D point clouds must be labeled. From a training and communication perspective, panoramic images and 3D point clouds must be integrated into environments compatible with visualization tools. For example, it would be necessary to integrate 3D point clouds into Unity to view them in virtual reality headsets. This dataset is licensed under CC BY-NC-SA 4.0.

## Ethics Statement

The authors have read and follow the ethical requirements for publication in Data in Brief and confirming that the current work does not involve human subjects, animal experiments, or any data collected from social media platforms.

## CRediT Author Statement

**Hervé Daudigny:** Methodology, Investigation, Validation, Writing – original draft, Data curation; **Pierre Grussenmeyer:** Methodology, Supervision, Writing.

## Declaration of Generative AI-assisted Technology

During the preparation of this work the authors used AI-assisted technologies to improve the readability and language of the manuscript. After using this tool/service, the authors reviewed and edited the content as needed and take full responsibility for the content of the published article.

## Data Availability

ZenodoFictional Forensic Scene (FFS) (Original data).
